# Outcomes of Patients Treated with Prehospital Noninvasive Ventilation: Observational Retrospective Multicenter Study in the Northern French Alps

**DOI:** 10.3390/jcm10071359

**Published:** 2021-03-25

**Authors:** Julie Pinczon, Nicolas Terzi, Pascal Usseglio-Polatera, Gaël Gheno, Dominique Savary, Guillaume Debaty, Vincent Peigne

**Affiliations:** 1 SAMU 73, Centre Hospitalier Métropole-Savoie, 73000 Chambéry, France; julie.pinczon@ch-metropole-savoie.fr (J.P.); pascal.usseglio-polatera@ch-metropole-savoie.fr (P.U.-P.); 2 Medical Intensive Care Unit, University Hospital of Grenoble Alps, 38000 Grenoble, France; nterzi@chu-grenoble.fr; 3 SAMU 74, Centre Hospitalier Annecy Genevois, 74370 Epagny-Metz-Tessy, France; ggheno@ch-annecygenevois.fr; 4 Emergency Department, CHU d’Angers, University Angers, 49000 Angers, France; dominique.savar@chu-angers.fr; 5 IRSET (Institut de Recherche en Santé, Environnement et Travail)—UMR_S 1085, University Angers, 49000 Angers, France; 6 Emergency Department, SAMU 38, University Hospital of Grenoble Alps and University Grenoble Alps/CNRS/TIMC-IMAG UMR 5525, 38000 Grenoble, France; gdebaty@chu-grenoble.fr; 7 Intensive Care Unit, Centre Hospitalier Métropole-Savoie, 73000 Chambéry, France

**Keywords:** noninvasive ventilation, pneumonia, COPD, acute pulmonary edema, prehospital investigation

## Abstract

Noninvasive ventilation (NIV) improves the outcome of acute cardiogenic pulmonary edema (AcPE) and acute exacerbation of chronic obstructive pulmonary disease (aeCOPD) but is not recommended in pneumonia. The aim of this study was to assess the appropriateness of the use of NIV in a prehospital setting, where etiological diagnostics rely mainly on clinical examination. This observational multicenter retrospective study included all the patients treated with NIV by three mobile medical emergency teams in 2015. Prehospital diagnoses and hospital diagnoses were extracted from the medical charts. The appropriateness of NIV was determined by matching the hospital diagnosis to the current guidelines. Among the 14,067 patients screened, 172 (1.2%) were treated with NIV. The more frequent prehospital diagnoses were AcPE (*n* = 102, 59%), acute respiratory failure of undetermined cause (*n* = 46, 28%) and aeCOPD (*n* = 17, 10%). An accurate prehospital diagnosis was more frequent for AcPE (83/88, 94%) than for aeCOPD (14/32, 44%; *p* < 0.01). Only two of the 25 (8%) pneumonia cases were diagnosed during prehospital management. Prehospital NIV was inappropriate for 32 (21%) patients. Patients with inappropriate NIV had a higher rate of in-hospital intubation than patients with appropriate NIV (38% vs. 8%; *p* < 0.001). This high frequency of inappropriate NIV could be reduced by an improvement in the prehospital detection of aeCOPD and pneumonia.

## 1. Introduction

Noninvasive ventilation (NIV) is recommended in the case of acute cardiogenic pulmonary edema (AcPE) and in the case of acute exacerbation of chronic obstructive pulmonary disease (aeCOPD) [1,2]. Studies have demonstrated with a high level of evidence that NIV decreases the intubation rates and mortality in these patients [3,4]. Most of these studies included inpatients. A meta-analysis identified 10 studies about NIV for the management of acute respiratory failure in the prehospital setting [5]. These studies involved 800 patients with AcPE, aeCOPD, pneumonia or acute respiratory failure of any cause. This meta-analysis suggested that prehospital NIV decreases the intubation and mortality rates, but the level of evidence was quite low. The benefits of prehospital NIV are better proven for AcPE than for other conditions [5,6,7,8,9]. Moreover, NIV failure is associated with worse outcomes in patients with de novo respiratory failure, such as pneumonia [9]. In the prehospital setting, differentiating the clear indications of NIV (AcPE and aeCOPD) from pneumonia may be challenging and could decrease the potential benefit of this therapy.

Prehospital NIV is widely available in France [10], although the only published epidemiological data about its use was gathered in the Paris area ten years ago [11].

The aim of this study was to assess the use of NIV in the prehospital setting according to the hospital diagnosis and to compare this with the current guidelines about NIV use.

## 2. Materials and Methods

### 2.1. Study Design and Population

We performed a retrospective observational study in 3 mobile medical emergency teams of the Northern French Alps: Annecy, Chambéry and Grenoble. Patients were included if they were aged >15 years and treated with prehospital NIV between 1 January 2015 and 31 December 2015.

According to the 2017 ERS guidelines [1], NIV was defined as the use of continuous positive airway pressure (CPAP) or intermittent positive pressure ventilation (IPPV, including pressure support ventilation (PS) or bilevel positive airway pressure ventilation (BiPAP)) with a noninvasive interface.

Relevant data, including the prehospital diagnosis and final diagnosis, duration of NIV, intubation and mortality, were extracted from prehospital and hospital charts.

### 2.2. Data Analysis

Qualitative data were represented as percentages and standard deviations (SD) and compared with the chi-square test. Quantitative data were represented as medians and interquartile ranges (IQR) and compared with nonparametric tests. All reported *p*-values were based on two-sided tests; a *p*-value of ≤0.05 was considered to indicate statistical significance. Data were analyzed with Statview (SAS Institute, Cary, North Carolina, USA) and R (R Foundation for Statistical Computing, Vienna, Austria) softwares.

### 2.3. Suitability of NIV Use with the Current Guidelines

Suitability of NIV use was assessed by the comparison of the initial diagnosis of the emergency medical services (EMS) physician with the final hospital diagnosis of each patient according to the French national guidelines [2]. French guidelines about NIV for acute respiratory failure were established in 2006. According to these guidelines, NIV must be performed in the case of AcPE and hypercapnic aeCOPD and should probably be used for immunocompromised patients with acute respiratory failure and in the case of blunt thoracic trauma and of acute exacerbation of chronic neuromuscular diseases. NIV should probably not be performed in the case of pneumonia and acute respiratory distress syndrome. The French guidelines are in accordance with the latest European Respiratory Society/American Thoracic Society guidelines [1], except for immunocompromised patients. International guidelines [1] do not recommend the use of NIV for the management of immunocompromised patients with acute respiratory failure. In this setting, some recent studies did not confirm the interest of NIV [12], even if a meta-analysis indicated with a low level of evidence that NIV could decrease the short term mortality and intubation rate [13].

In France, prehospital emergency medical services (EMS) consist of mobile intensive care units staffed by a paramedic driver, a nurse and a senior emergency physician. Mobile intensive care units are dispatched in the case of respiratory distress with severity criteria (unable to speak, respiratory rate >30/min and oxygen saturation <90%) and provide high-intensity care like tracheal intubation, mechanical ventilation, NIV with face masks, vasopressors and thoracostomy. The participating prehospital EMS could not provide high-flow nasal cannula (HFNC) during the study period. Mild respiratory distresses without severity criteria are usually transported to the emergency department with paramedics only (firefighter team or ambulance) without NIV capacity. Only patients managed with mobile intensive care units were included. As prehospital blood gas analyses were never performed (unavailable), we considered that all aeCOPD patients had prehospital hypercapnia. Prehospital lung ultrasonography was not available during the study period. Prehospital diagnoses were based on the clinical judgment and physiological assessment of the attending physician.

## 3. Results

### 3.1. Study Population

The three participating mobile medical emergency teams managed 14,067 patients during the study period (Grenoble 6123 patients, 43.5%, Annecy 4150, 29.5% and Chambéry 3794, 27%), and 172 (1.2%) patients received NIV. NIV was more frequently used at Chambéry (72/3794, 2.0%) than at Annecy (42/4150, 1.0%) and Grenoble (58/6123, 0.95%; *p* < 0.001). Prehospital charts were available for all these 172 patients.

One patient was left on the scene. Hospital charts were available for 155 (91%) of the 171 patients admitted into a hospital.

### 3.2. Prehospital NIV

Among the 172 patients treated by NIV (57% men, median age 78, IQR 66–85), 10 (6%) had home oxygen therapy and 14 (8%) had home long-term NIV. Only one patient, an 89-year-old man with home long-term NIV, had a do-not-intubate order.

Indications of NIV, described in Table 1, were mostly AcPE (*n* = 102, 59%), acute respiratory failure of undetermined cause (*n* = 46, 28%) and aeCOPD (*n* = 17, 10%).

The median duration of case management by the mobile medical emergency team was 60 min (IQR 50–75). IPPV ventilation was applied to 132 (77%) patients and CPAP to 40 (23%). As shown in Table 1, all the patients treated with CPAP had a prehospital diagnosis of AcPE. The median inspired fraction of oxygen was 70%. The median maximal positive end expiratory pressure was 6 cm H_2_O, and the median positive inspiratory pressure was 16 cm H_2_O.

A prehospital failure to use NIV, leading to a prehospital interruption of NIV, was reported in nine (5%) cases: one patient who had CPAP for AcPE, three patients who had IPPV for AcPE, one patient who had IPPV for aeCOPD and four patients who had IPPV for acute respiratory failure of undetermined cause during the prehospital management. The hospital diagnoses of these four patients were aeCOPD for three of them and pneumonia for the other one. The failure rate was 2.5% (1/40) for CPAP and 6.0% (8/132) for IPPV (*p =* 0.69).

During the prehospital management, three (1.7%) patients were intubated (two with aeCOPD treated with IPPV and one with AcPE treated with CPAP). One (0.6%) patient, the one with AcPE who was intubated, died in the prehospital setting (cardiac arrest following intubation).

### 3.3. Hospital Outcome

During the hospital stay of the 155 patients with available hospital data, 84 (54%) patients had at least one supplementary session of NIV, 26 (17%) were intubated, 9 (6%) had HFNC and 57 (37%) died.

NIV was resumed for all 14 patients who already had long-term NIV, and 10 of them (71%) died. Among the 70 other patients who had NIV again at the hospital, 39 patients (10 intubations and seven deaths, including three deaths after intubation) had NIV for less than 24 h, 7 patients (one intubation and three deaths with two death after intubation) had a duration of NIV between 24 and 48 h and 13 patients (seven deaths, including the four patients who were intubated) received NIV during 48 to 96 h. For one patient, NIV was stopped at the 30th day. The duration of NIV could not be extracted from the charts of the nine remaining patients.

The diagnoses retained at hospital discharge and the main outcomes are described in Table 2.

### 3.4. Comparison of Prehospital and Hospital Diagnoses

Table 3 compares the prehospital and final diagnoses for the 155 patients analyzed.

The rate of accurate prehospital diagnosis was higher for AcPE patients (83/88, 94%) than for aeCOPD (14/32, 44%, *p* < 0.01). Pneumonia was diagnosed during the prehospital management for only two of the 25 (8%) of the patients who had pneumonia as the final diagnosis.

### 3.5. NIV Use and Suitability with the National Guidelines

As shown in Table 2 and Table 4, 123 patients (79%) received prehospital NIV in agreement with the guidelines, and 32 (21%) patients should probably not have been treated with NIV according to the guidelines.

NIV was not recommended for these 32 patients, because 25 (78%) of them had pneumonia, 2 had pneumothorax (one after blunt trauma and one spontaneous), 2 had poisoning, one had primary pulmonary arterial hypertension, one had an acute respiratory failure of an immunocompromised patient, one had an acute respiratory distress syndrome related to an acute pancreatitis and the last one had encephalitis. Among these 32 patients, NIV was changed for a high-flow nasal cannula for eight (25%) patients (all with pneumonia) and was resumed for 18 (56%) patients, for more than two days for six (33%) of them. Twelve (38%) patients were intubated, including seven of the 18 (39%) patients with a prolongation of nonrecommended NIV and five of the eight patients (63%) with HFNC. Eleven (34%) patients with nonrecommended NIV died.

The 123 (79%) patients who had NIV with respect to the guidelines had a similar rate of NIV continuation (66, 54%; *p =* 0.79), had less HFNC (one patient with aeCOPD, <1%; *p* < 0.001), less intubation (10, 8%; *p <* 0.001) and a similar mortality (45, 37%; *p =* 1) than the patients with nonrecommended NIV.

## 4. Discussion

This multicenter retrospective observational study assessed the relevance of the “real life” use of prehospital NIV in the French Northern Alps. Its major strength was to bridge the gap between prehospital and hospital conditions by comparing prehospital and final diagnoses. Among our study population, NIV was an uncommon therapy in prehospital settings (1% of the patients), and only 172 patients were investigated, although more than 14,000 were screened. Interestingly, NIV should not have been proposed to 21% of the analyzed patients, and half of them resumed NIV at the hospital. Patients with unsuitable NIV had a higher rate of intubation than patients with adequate NIV.

The impact on the outcome of the inappropriate NIV was not evaluated, as the study focused on NIV and not acute respiratory failure. Although we may wonder if it would have been beneficial for these patients to be intubated earlier or to have been treated more frequently with HFNC, the higher rate of intubation among the patients with inappropriate NIV in the present study was more probably related to the cause of their acute respiratory failure rather than to their prehospital management. Nevertheless, NIV failure has been associated with a worse outcome in such patients [9]. Our data do not support an association between inappropriate NIV and mortality. On the contrary, HFNC is a promising therapy for hypoxemic pneumonia, with the recent data suggesting that it could decrease the need for tracheal intubation [12]. NIV and HFNC were not associated with an improvement in the outcome of patients with acute hypoxemic respiratory failure [14]. Although the NIV appropriateness was evaluated according to the current French guidelines [2], the conclusions of the present study can be generalized to other settings, because these recommendations are in accordance with the latest international guidelines [1].

Some limits had to be considered in our study. First, we were not able to assess the proportion of patients with respiratory distress that could have benefitted from NIV, according to the French guidelines. Nevertheless, the proportion of patients treated with NIV had the same magnitudes in the three participating centers, and this study reflected the current practice during that period. Second, several data are missing due to incomplete files.

Pneumonia, which should preferentially be treated by HFNC [15] and not by NIV [1,2], was the leading condition (78%) associated with inappropriate prehospital NIV in our study and was frequently unrecognized during prehospital management. On the contrary, 91% of the prehospital diagnoses of AcPE were accurate among our patients. Less than half of the aeCOPD, for whom prehospital NIV is highly profitable [8], were identified before hospital admission in the present study. These findings point out how difficult it can be to investigate acute respiratory failure in a prehospital setting.

As uncertainty is a common pitfall in the prehospital setting, improving the accuracy of prehospital diagnoses appears to be the current challenge to improve prehospital NIV use. According to our results, the two main axes are the detection of aeCOPD and of pneumonia. Point-of-care arterial blood gases measurement is precious for physiological assessments and could identify hypercapnic patients, who are more susceptible to aeCOPD than normocapnic patients [16]. A noninvasive alternative is the measurement of transcutaneous CO_2_ partial pressure, which is a validated surrogate of arterial CO_2_ partial pressure during aeCOPD [17]. These methods would allow excluding normocapnic aeCOPD patients who do not need NIV [1,2,12]. Another promising tool is point-of-care echography: patients with sonographic patterns of pneumonia could be identified and be denied as having NIV [18]. The feasibility of prehospital lung echography has been reported, with encouraging results for AcPE detection by physicians [19].

## 5. Conclusions

Prehospital NIV was inappropriate in 21% of cases, and these patients had a higher rate of intubation than patients with a validated indication of NIV. This high frequency of inappropriate NIV can be explained by the difficulty to investigate acute respiratory failure in the prehospital setting and could be reduced by an improvement of the prehospital detection of aeCOPD and pneumonia.

## Figures and Tables

**Table 1:** Prehospital data of the 172 patients treated with noninvasive ventilation.

Prehospital Diagnosis	*n* (%)	Age (Years)	CPAP (*n*, %)	IPPV (*n*, %)	FiO2 (%)	PEEP Max (cm H_2_O)	PIP Max (cm H_2_O)	NIV Failure (*n*, %)
Acute cardiogenic pulmonary edema	102 (59%)	81 (72–87)	40 (39%)	62 (61%)	80% (50–100)	6 (5–8)	16 (15–18)	4 (4%)
Acute respiratory failure of undetermined cause	46 (28%)	71 (62–80)	0	46 (100%)	60% (40–95)	5 (5–5)	16 (14–17)	4 (9%)
Acute exacerbation of chronic obstructive pulmonary disease	17 (10%)	72 (64–82)	0	17 (100%)	40% (25–60)	6 (5–6)	19 (16–21)	1 (6%)
Pneumonia	4 (2%)	67 (55–80)	0	4 (100%)	75% (45–100)	8 (8–12)	13 (12–13)	0
Poisoning	2 (1%)	64–73	0	2 (100%)	30%	4–8	10–20	0
Blunt trauma	1 (<1%)	18	0	1 (100%)	50%	5	12	0

Quantitative data are expressed as the median and interquartile range, except for the “poisoning” and “trauma” lines where individual data are mentioned. CPAP: Continuous Positive Airway Pressure, IPPPV: Intermittent Positive Pressure Ventilation, NIV: Noninvasive Ventilation, PEEP: Positive End Expiratory Pressure, PIP: Positive Inspiratory Pressure, FiO2: Fraction of Inspired Oxygen.

**Table 2:** Hospital outcomes of the 155 patients with prehospital noninvasive ventilation and the available hospital data.

Final Diagnosis	*n* (%)	Age	Adequate Use of NIV	Hospital NIV	Hospital Intubation	Hospital Mortality
Acute cardiogenic pulmonary edema	88 (57%)	82 (73–87)	YES	39 (44%)	6 (7%)	28 (32%)
Acute exacerbation of chronic obstructive pulmonary disease	32 (21%)	75 (63–83)	YES	26 (81%)	4 (13%)	16 (50%)
Pneumonia	25 (16%)	73 (67–81)	NO	11 (44%)	15 (60%)	9 (36%)
Pneumothorax	2 (1%)	18–82	NO	2 (100%)	0	1 (50%)
Neuromuscular diseases	2 (1%)	37–72	YES	1 (50%)	0	1 (50%)
Poisoning	2 (1%)	52–64	NO	2 (100%)	0	0
Pulmonary arterial hypertension	1 (<1%)	66	NO	1 (100%)	0	0
Acute respiratory failure of an immunocompromised patient	1 (<1%)	39	NO	0	0	0
Acute respiratory distress syndrome of extra-thoracic cause	1 (<1%)	71	NO	1 (100%)	0	1 (100%)
Encephalitis	1 (<1%)	75	NO	1 (100%)	1 (100%)	1 (100%)

Quantitative data are expressed as the median and interquartile range in the first 3 lines. Individual data are mentioned in the other lines.

**Table 3:** Comparison of the prehospital and final diagnoses of the 155 patients with prehospital noninvasive ventilation and the available hospital data.

Prehospital Diagnosis (*n*)	Final Diagnosis (*n*, %)
Acute cardiogenic pulmonary edema (91)	Acute cardiogenic pulmonary edema (84/91, 91%)
	Pneumonia (4/91, 4%)
	Acute exacerbation of chronic obstructive pulmonary disease (3/91, 3%)
	Pulmonary arterial hypertension (1/91, 1%)
Acute exacerbation of chronic obstructive pulmonary disease (16)	Acute exacerbation of chronic obstructive pulmonary disease (14/16, 88%)
	Pneumonia (1/16, 6%)
	Pneumothorax (1/16, 6%)
Acute respiratory failure of undetermined cause (43)	Pneumonia (17/43, 40%)
	Acute exacerbation of chronic obstructive pulmonary disease (15/43, 35%)
	Acute cardiogenic pulmonary edema (5/43, 11%)
	Miscellaneous (6/43, 14%)
Pneumonia (2)	Pneumonia (2/2, 100%)
Poisoning (2)	Pneumonia (1/2, 50%)
	Opioid poisoning (1/2, 50%)
Blunt trauma (1)	Pneumothorax (1/1, 100%)

**Table 4:** Outcome of the patients with NIV indicated and not recommended according to the guidelines.

Data	NIV Indicated (*n*, %)	NIV Not Recommended (*n*, %)	*p*-Value
*n*	123	32	
NIV continuation	66 (54%)	18 (56%)	0.79
High flow nasal cannula	1 (<1%)	8 (25%)	<0.001
Intubation	10 (8%)	12 (38%)	0.001
Hospital mortality	45 (37%)	11 (34%)	1

## Data Availability

Data available on request due to restrictions.
